# Continuous vital sign monitoring of acute Lassa fever using wearable biosensor devices in West Africa

**DOI:** 10.1038/s43856-025-01002-6

**Published:** 2025-07-11

**Authors:** Brady Page, Raphaëlle Klitting, Matthias G. Pauthner, Steven Steinhubl, Stephan Wegerich, Margaret Kaiser, Foday Alhasan, Edwin Konuwa, Veronica Koroma, Ibrahim Sumah, Jenneh Brima, Tiangay Kallon, Brima Jusu, Sia Mator-Mabay, Mohamed Kamara, Fatima Kamara, Emilia Jaward, Angella Massally, Zainab Kanneh, Michelle McGraw, John Schieffelin, Donald Grant, Kristian G. Andersen

**Affiliations:** 1 University of California, San Diego (UCSD), La Jolla, CA USA; 2 Scripps Research Institute, La Jolla, CA USA; 3 Prolaio, Inc, Scottsdale, AZ USA; 4 Independent researcher, Greenville, SC USA; 5 Kenema Government Hospital, Kenema, Sierra Leone; 6 Tulane University School of Medicine, New Orleans, LA USA

**Keywords:** Viral infection, Cardiac device therapy

## Abstract

**Background:**

Lassa fever is a fulminant viral illness associated with high in-hospital mortality. This disease constitutes a serious public health concern in West Africa, in particular Nigeria and the Mano River Union region (Guinea, Liberia, and Sierra Leone). In Sierra Leone, continuous monitoring of critically ill patients is hindered by a lack of equipment and personnel.

**Methods:**

We used wearable biosensor devices to remotely monitor hospitalized individuals with acute Lassa fever in order to describe vital sign trends that may be associated with clinical outcome and to evaluate the feasibility of this approach in a resource-limited setting.

**Results:**

The case fatality rate among participants (*n* = 8) was 62.5%, with a median time from hospital presentation to death of 2 days. Our results show that non-survivors (*n* = 5) spent a greater proportion of their monitoring period in the age-specific tachycardia range (45.8%) compared to survivors (1.7%), and had lower mean heart rate variability (10 ms) compared to those that survived (59 ms). Due to inconsistent sensor adhesion, as well as Bluetooth and cellular connectivity issues, over 80% of collected vital sign data was discarded for poor quality.

**Conclusions:**

Real-time monitoring of vital signs using wearable biosensors may have the potential to identify individuals at increased risk for poor outcomes in Lassa fever by detecting age-specific tachycardia and reductions in heart rate variability. Whether this represents an improvement upon traditional bedside vital sign collection in resource-limited settings is not clear, as a substantial proportion of monitoring data was of poor quality. Technical improvements in sensor connectivity and adhesion are needed to enable widespread use of this device, for both clinical and research purposes.

## Plain language summary

Lassa fever is a viral illness in West Africa that is associated with high rates of death among individuals admitted to the hospital for treatment. Monitoring of critically ill patients within the infection’s geographic range is hindered by a lack of equipment and personnel. To better understand vital sign trends and to explore different approaches to patient monitoring, wearable biosensors were used to monitor patients with Lassa fever in Sierra Leone. We found that tachycardia and reduced heart rate variability are associated with poor outcomes, though technical limitations hinder broader implementation of such devices within regions where Lassa fever is present.

## Abstract (web summary)

Page et al., use wearable biosensors to monitor vital sign trends in patients with Lassa fever in Sierra Leone. Tachycardia and reduced heart rate variability associate with poor outcomes, though technical limitations hinder broader implementation.

## Introduction

Lassa fever is a fulminant systemic viral illness that is endemic to West Africa and is associated with seasonal outbreaks of high mortality^1^. Though the establishment of accurate figures has been challenging, it is estimated that the infection is responsible for 300,000 infections and 5,000 deaths annually, with in-hospital mortality rates ranging from 20 to 60%^2^. A major driver of mortality in Lassa fever is multiple organ failure from hemodynamic collapse^3^. Sierra Leone, one of the world’s poorest and least developed countries, generally lacks the material and nursing resources to frequently and safely monitor patients with Lassa fever while maintaining rigorous containment measures^4,5^. Despite a transient influx of healthcare infrastructure during the 2014–2015 ebola outbreak, an alarming deficit in staffing remains^6^.

Previous efforts to augment monitoring and management capacity for critically ill patients infected with high-consequence pathogens in low- and middle-income countries have employed innovative new technologies with the potential to economically and reliably begin to address this deficit^7,8^. Mobile health technologies–such as wearable biosensor devices for continuous vital sign monitoring–have the advantage of minimizing exposure to infectious patients while providing an abundance of objective data without much overhead or expertise, compared to more durable telemetry systems^9,10^. The promise of wearable sensors is that the early detection of certain physiological trends can warn clinicians about patients at risk for deterioration and lead to earlier intervention^11^. These devices have repeatedly been validated for remote vital signs monitoring and mortality prediction among unstable patients in low-resource settings, including during the 2014–2016 outbreak of Ebola virus disease in Sierra Leone^7,12–14^.

In this study, we use wearable biosensor devices to continuously and remotely monitor individuals with Lassa fever in an under-resourced endemic area of Sierra Leone, to describe vital sign changes that accompany clinical outcomes and to evaluate the feasibility and efficacy of this approach in such a setting. We find that monitoring of vital signs using wearable biosensors has the potential to identify individuals at increased risk for poor outcomes in Lassa fever by detecting age-specific tachycardia and reductions in heart rate variability, though technical improvements in sensor connectivity and adhesion are needed to enable widespread application of this device.

## Methods

### Study design and setting

This prospective observational study took place in 2019–2021 at Kenema Government Hospital (KGH), the national treatment center for Lassa fever in Sierra Leone where all identified and suspected cases of the disease are transferred. KGH is a secondary referral center with a large catchment area that includes both urban and rural settings in the country’s Eastern Province.

### Patient selection and continuous physiological monitoring

**Fig. 1:**
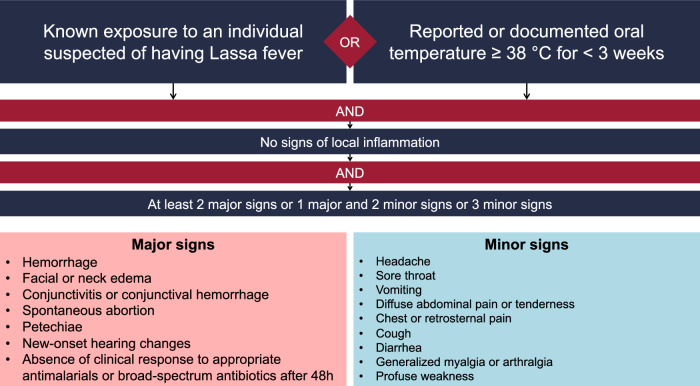
Lassa fever case definition criteria.

**Fig. 2:**
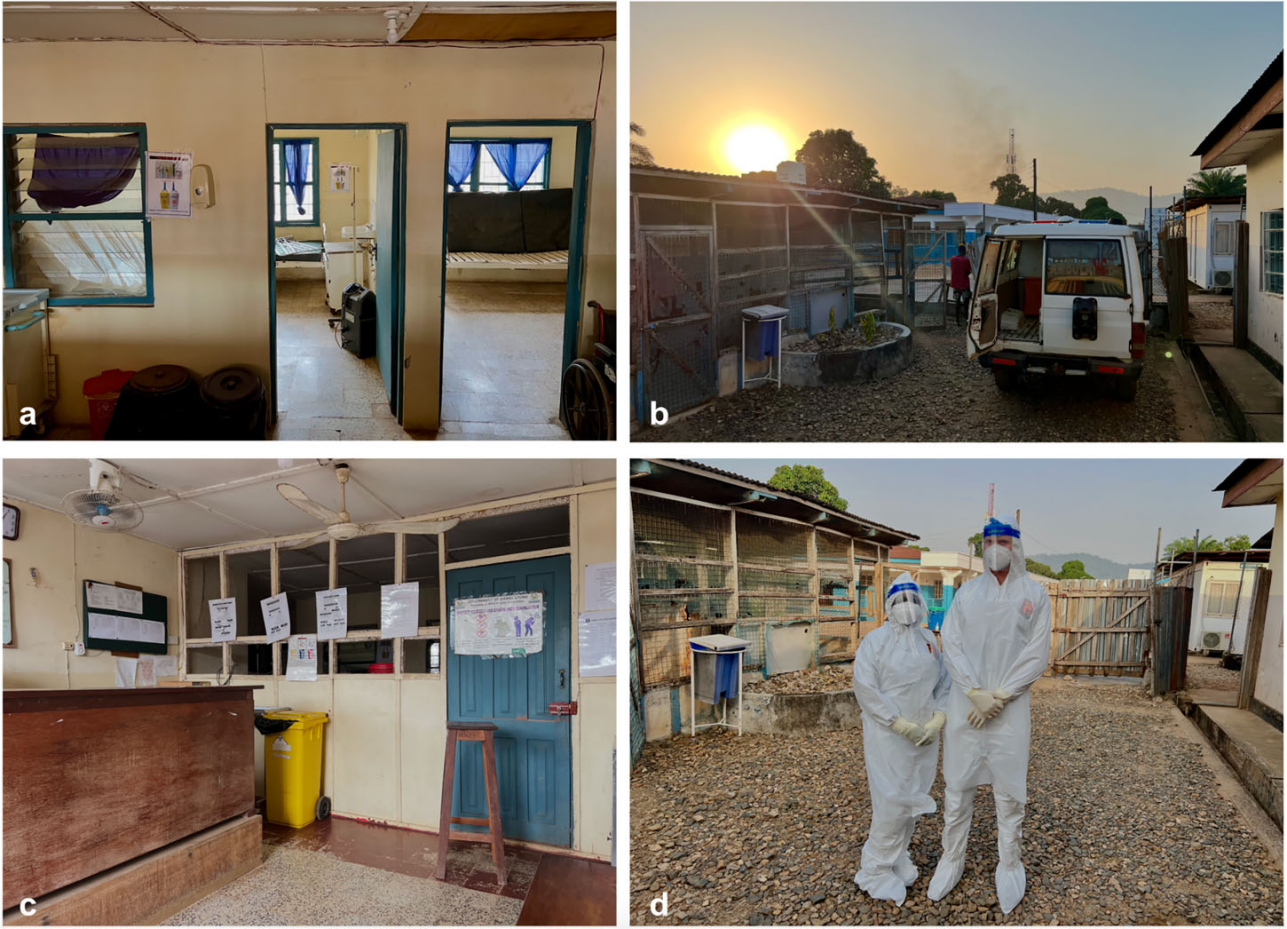
The Kenema Government Hospital Lassa fever ward. The KGH Lassa fever ward is a freestanding building in an isolated corner of the hospital grounds. Each patient is cared for in one of eleven individual rooms (**a**), all of which are connected by a single hallway leading from the outdoor donning area to the outdoor doffing area. Patients arrive from other units of the hospital or from the community via ambulance, which is parked in a dedicated ambulance bay (**b**). Specialized Lassa fever nurses are based at the nurses’ station (**c)** outside of the biocontainment section of the ward, from where patients can be visualized through a row of windows that are situated ten feet from the nearest patient rooms. Prior to entering the isolation area, physicians and nurses don personal protective equipment (**d)** and follow biocontainment standards as recommended by World Health Organization guidelines.

Individuals of all ages admitted to KGH with acute Lassa fever between January 2019 and July 2021 were invited to enroll. Inclusion criteria were a clinical presentation that satisfied the case definition criteria for Lassa fever (Fig. 1) and a positive serum antigen test for Lassa virus (Zalgen Labs, Frederick, MD, USA). Exclusion criteria included a history of allergy to skin adhesive. All confirmed cases were cared for in a designated Lassa ward with enhanced biocontainment measures, as outlined by World Health Organization guidelines (Fig. 2)^15,16^. The KGH Lassa ward has 24-h specialized nursing that performs in-person patient assessments every 6 h for medication administration, specimen collection for diagnostics, and vital sign collection via oral thermometer, manual sphygmomanometer, and digital pulse oximeter. Vital sign collection, diagnostic testing, and treatment were conducted throughout admission to the local standard of care, which includes a daily evaluation by the treating physician. All patients admitted to the Lassa ward underwent rapid testing for malaria, received antibiotics as determined by the treating physician, and began treatment with intravenous ribavirin given as a 30 mg/kg loading dose, followed by 15 mg/kg every 6 h for 4 days and 7.5 mg/kg every 6 h for 6 more days.

Upon admission to the Lassa ward, a battery-powered VitalPatch wearable biosensor device (VitalConnect, San Jose, CA, USA) was applied to each patient’s chest by a healthcare worker in full personal protective equipment (PPE). The device is an FDA-approved, 115 × 36 × 8 mm, 11 g, battery-powered, flexible strip with an adhesive side that attaches to the skin of a patient’s chest. Devices were linked via Bluetooth to Android devices running the physIQ data analytics platform (physIQ, Chicago, IL, USA), which were located in a nursing station outside of the isolation area. Given its observational nature, continuous vital signs collected as part of the study were not available to influence clinical care. All nurses received training in device placement, use of the analytic application, study procedures, and research ethics. Devices were worn until discharge from the Lassa ward, which was considered once patients had clinically improved, completed a 10-day course of ribavirin, and produced a negative serum antigen test for Lassa virus. If a device’s battery expired prior to discharge or there were issues with adhesion, a new device was applied.

### Physiological analysis

Waveform data from the EKG leads, thermistor, and accelerometer present on biosensor devices were analyzed by the machine learning algorithms of the physIQ platform to generate the following parameters at 1-min intervals: skin temperature, heart rate (HR), respiratory rate (RR), time domain heart rate variability (HRV), and EKG signal quality. The physIQ algorithm makes calls to the biosensor at 1-min intervals via a Python middleware script that allows for the collection of biosensor data, as previously described^12,17^. The algorithm is applied to the EKG signal to assess the signal’s strength, motion artifact content, and noise levels over the 1-min intervals. This results in a quality metric that ranges from 0 to 100% for each 1-min data window. If the metric falls below 75%, the corresponding 1-min data window is automatically rejected for use in calculating vital signs from the raw biosignal data.

### Quality control

As a quality control measure, and since the focus of the study was on physiological trends throughout the course of Lassa fever hospitalization, we established standards for the EKG signal quality metric and vital sign values. In addition to the automatic filtering of patient physiological data to remove 1-min data windows where EKG quality was deemed by the machine learning algorithms to be less than 75%, we also filtered 1-min data windows during which vital sign readings were physiologically unlikely–namely, skin temperature <33 °C or > 42 °C, or respiratory rate of 0. Patients who did not produce at least 12 h of continuous high-quality physiological monitoring data were not included in the analysis.

### Statistical analysis

Descriptive statistics were used to summarize patient demographics and vital signs. Testing for statistical significance was not performed due to the study’s small sample size.

### Ethics statement

Written informed consent was obtained from all participants or, if an individual was unable to provide informed consent, from an appropriately identified surrogate. Written informed consent was obtained from the parent or guardian of each participant under 18 years of age. This study was approved by research ethics boards at Tulane University (IRB #140674) as well as the Sierra Leone Ethics and Scientific Review Committee (SLESRC No: 022/09/2022).

### Reporting summary

Further information on research design is available in the Nature Portfolio Reporting Summary linked to this article.

## Results

Thirty patients were admitted to the KGH Lassa ward during the study period and seventeen were enrolled in this study. Nine individuals (52.9%) were excluded for not yielding at least 12 h of continuous physiological monitoring data meeting the study’s quality standards, leaving eight participants to be included in the analysis. Discontinuous monitoring was due to both a lack of adhesion of the device, as well as Bluetooth and cellular connectivity issues. Of the nine excluded patients, one participant died before the sensor could be placed, and three participants had their monitors placed but died within the next 20 h. There was no difference in the mortality rate between included and excluded participants, though excluded patients died sooner after presentation and enrollment (Supplementary Table 1**. Demoographic, clinical, and monitoring data for included and excluded individuals**). Among the eight patients included, the median age was 6.5 years (0.4–40 years), 50% were female, the median time from onset of symptoms to admission was 7 days (2–21 days), and rapid malaria test was positive in 50% of participants. The in-hospital mortality rate for all eligible patients during the study period was 64.7% and 62.5% among those included in the analysis, with a median time from enrollment to death of 32 h among all patients and 45 h among those included in the analysis. Patient demographics, presenting symptoms, vital signs on admission, and clinical outcome are presented in Table 1.

**Table 1:** Baseline demographics and vital signs of participants on admission to the Lassa ward

Pt	Sex	Age	Presenting symptoms	Days of symptoms	Temp	HR	BP	RR	SpO2	Malaria testing	Antibiotics	Antimalarials	Outcome
1	M	0–5	Fever, vomiting, diarrhea, weakness, dizziness, jaundice.	8	38.3	126	93/59	32	96%	Positive	Ceftriaxone, metronidazole	Yes	Survived
2	M	30–35	Fever, headache, sore throat, dizziness, retrosternal pain, myalgia, arthralgia.	7	37.9	61	123/71	22	97%	Negative	Ceftriaxone, metronidazole	Yes	Survived
3	M	40–45	Fever, weakness, dizziness, conjunctival injection, hiccups, vomiting, headache, myalgia, arthralgia, sore throat.	5	37.2	74	111/60	20	94%	Negative	Ceftriaxone	Yes	Survived
4	F	20–25	Fever, headache, vomiting, weakness, dizziness, seizure, confusion, abdominal pain.	2	37.7	116	125/77	24	91%	Negative	Ceftriaxone, metronidazole	No	Deceased
5	F	0–5	Fever, epistaxis, gingival bleeding, hematemesis, sore throat, cough, vomiting, weakness.	21	37.4	157	-	86	50%	-	Ceftriaxone, ampicillin, gentamicin	No	Deceased
6	M	0–5	Fever, bleeding from injection sites, petechiae, headache, vomiting, cough, diarrhea, weakness.	7	39.6	193	-	56	89%	Positive	Ceftriaxone, ampicillin	Yes	Deceased
7	F	6–10	Fever, gingival bleeding, hematemesis, bleeding from injection sites, seizure, headache, sore throat, cough, diarrhea, vomiting, jaundice, abd pain.	4	37.8	113	-	42	92%	Positive	Ceftriaxone, metronidazole	Yes	Deceased
8	F	0–5	Fever, seizure, sore throat, cough, weakness, vomiting, abdominal pain.	8	36.8	81	-	30	100%	Positive	Cefuroxime	Yes	Deceased

Vital signs were collected manually on admission to the Lassa ward. Age is in years, temperature is in °C, heart rate (HR) is in beats per minute, blood pressure (BP) is in mmHg, respiratory rate (RR) is in breaths per minute, and peripheral oxygen saturation (SpO2) is expressed as a percentage. Days of symptoms are those prior to admission. Antibiotics are only those begun on admission.

**Fig. 3:**
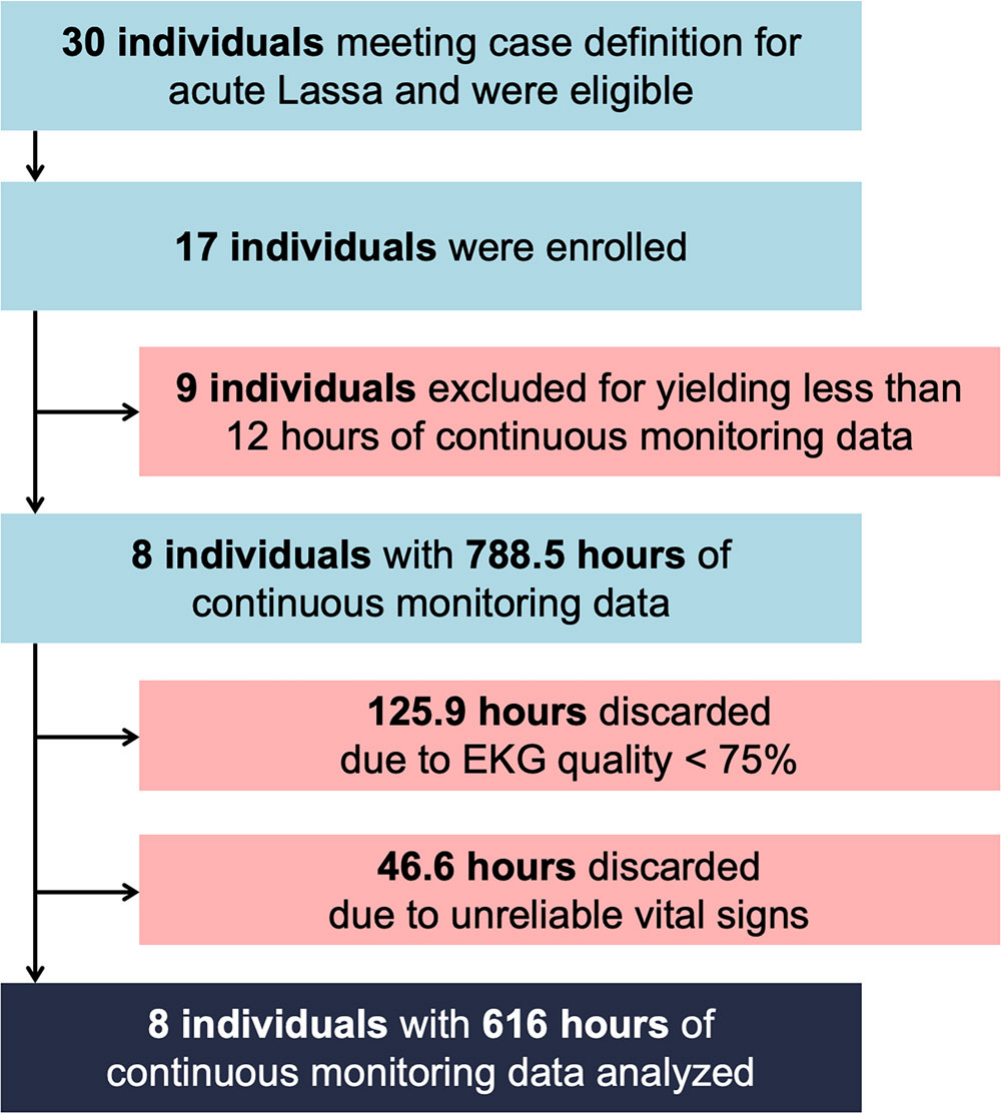
Flow chart of individuals and total hours excluded from analysis. A total of 30 patients were admitted to the KGH Lassa ward during the study period and enrolled in our study to be continuously monitored. Twenty-two individuals were excluded for yielding <12 h of continuous physiological monitoring data. Among the 8 participants included in the study, a total of 788.5 h of continuous waveform data were collected, with 172.5 h discarded for poor quality, defined as EKG quality <100% or or spurious vital signs, which were defined as respiratory rate of 0 or skin temperature that was either <33 °C or > 42 °C. Ultimately, 616 h of waveform data were analyzed.

**Fig. 4:**
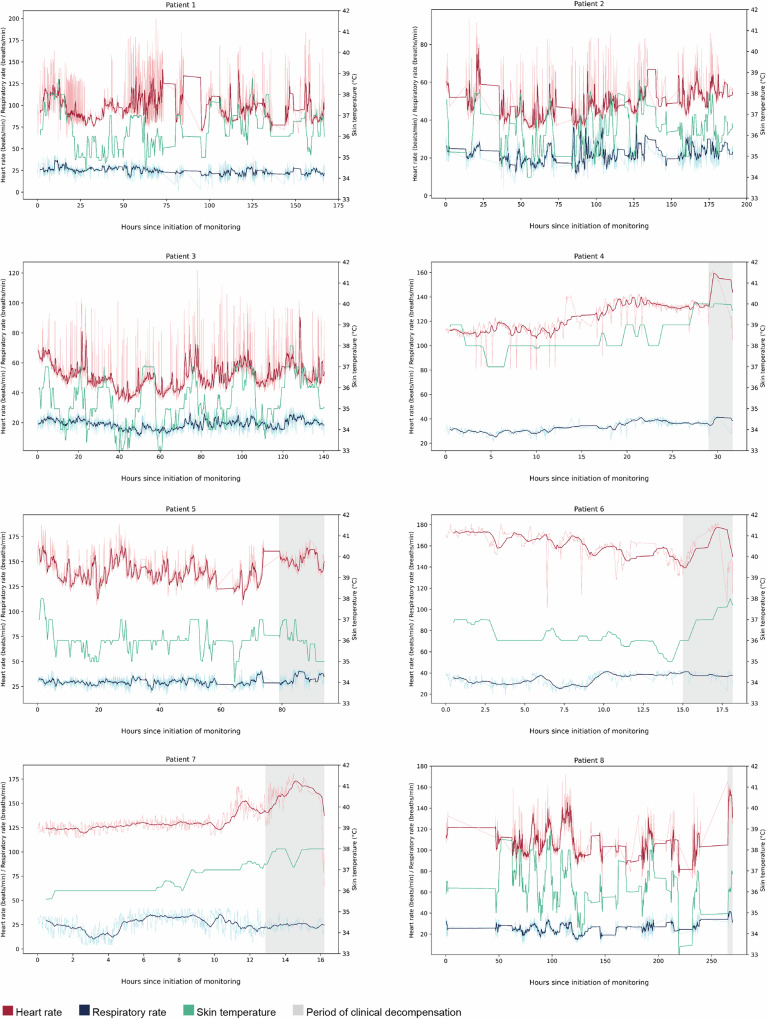
Heart rate, respiratory rate, and skin temperature during the course of acute Lassa fever. Trends are displayed for all eight individuals that were included in the analysis. The dark red and dark blue curves represent the 30-min rolling averages for HR and RR, respectively, while the light red and light blue curves represent HR and RR at 1-min intervals, as they were recorded by the device. Skin temperature is displayed as a 30-min rolling average.

Among the eight participants included in the analysis, a total of 788.5 h of continuous waveform data were collected, with 172.5 h (21.9%) discarded for poor quality (Fig. 3). Ultimately, 616 h of waveform data were analyzed, with an average of 77 h (12.7–151.7 h) per patient (Fig. 4). Four (50%) participants required the application of a new biosensor during their hospitalization: one when the battery on their first device expired and three more when there were issues with the device’s adhesive surface (Table 2). There were no adverse reactions to the devices.

**Table 2:** Features of continuous remote monitoring

Patient	Total time of continuous monitoring	Time of continuous monitoring analyzed	Time until new device applied	Reason for new device
1	128.5	79.5	108	Adhesion
2	154.0	118.0	73	Adhesion
3	139.3	133.3	120	Battery
4	31.6	26.3	-	-
5	87.4	78.9	-	-
6	22.9	12.7	-	-
7	17.1	15.6	-	-
8	207.7	151.7	137, 70	Adhesion
**Total**	**788.5**	**616.0**		

Time is reported in hours.

**Fig. 5:**
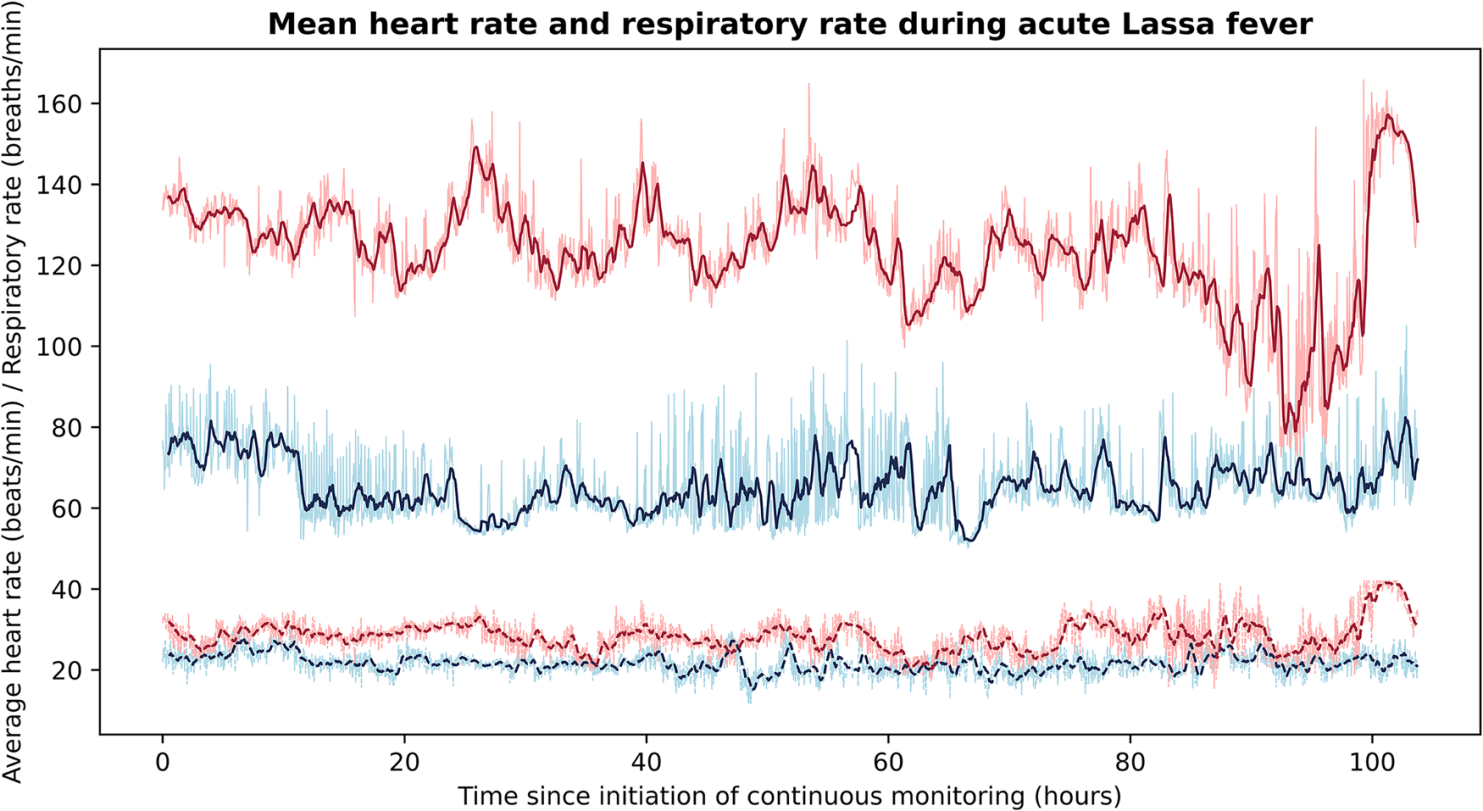
Mean heart rate and respiratory rate during acute Lassa fever among survivors and the deceased. The dark blue and dark red curves represent the 30-minute rolling averages of vital signs for survivors (*n* = 3) and the deceased (*n* = 5), respectively, while the light blue and light red curves report vital signs at 1-min intervals. Solid lines represent HR and dashed lines represent RR. Among those that survived acute Lassa fever (*n* = 3), mean heart rate (HR) and respiratory rate (RR) were lower than those of those who died (*n* = 5) at nearly all time points over the first 104 h of continuous monitoring. The mean HR and RR for survivors were 63 beats per minute and 22 breaths per minute, respectively, and 126 beats per minute and 29 breaths per minute for those who died.

Among those that survived acute Lassa fever (*n* = 3), mean HR and RR were lower than that of those who died (*n* = 5) at nearly all time points during the continuous monitoring period (Fig. 5). The mean HR and RR for survivors were 63 beats per minute and 22 breaths per minute, respectively, and 126 beats per minute and 29 breaths per minute for those that died, though these results are likely confounded by age. To account for age differences among our cohort–which was composed of both children and adults–we compared study HR and RR to established age-specific normal resting values. Age-specific thresholds for tachycardia and tachypnea were defined as the 90th percentile of published resting HR and RR data for a given age, respectively^18,19^. For each of the three survivors, mean HRs were below the age-specific thresholds for tachycardia; however, mean HRs were in the tachycardic range for four of the five non-survivors, whose HRs were on average 9.4% higher than each’s age-specific threshold for tachycardia. Overall, survivors spent 1.7% of their monitoring period in the age-specific tachycardic range, while non-survivors spent 45.8% of their monitoring period in the age-specific tachycardic range. Only one of the three survivors had a mean RR above the age-specific thresholds for tachypnea, while this was true for two of the five non-survivors. Survivors spent 40.7% of their monitoring period in the age-specific tachypneic range, while non-survivors spent 23.1% of their monitoring period in the age-specific tachypneic range.

**Fig. 6:**
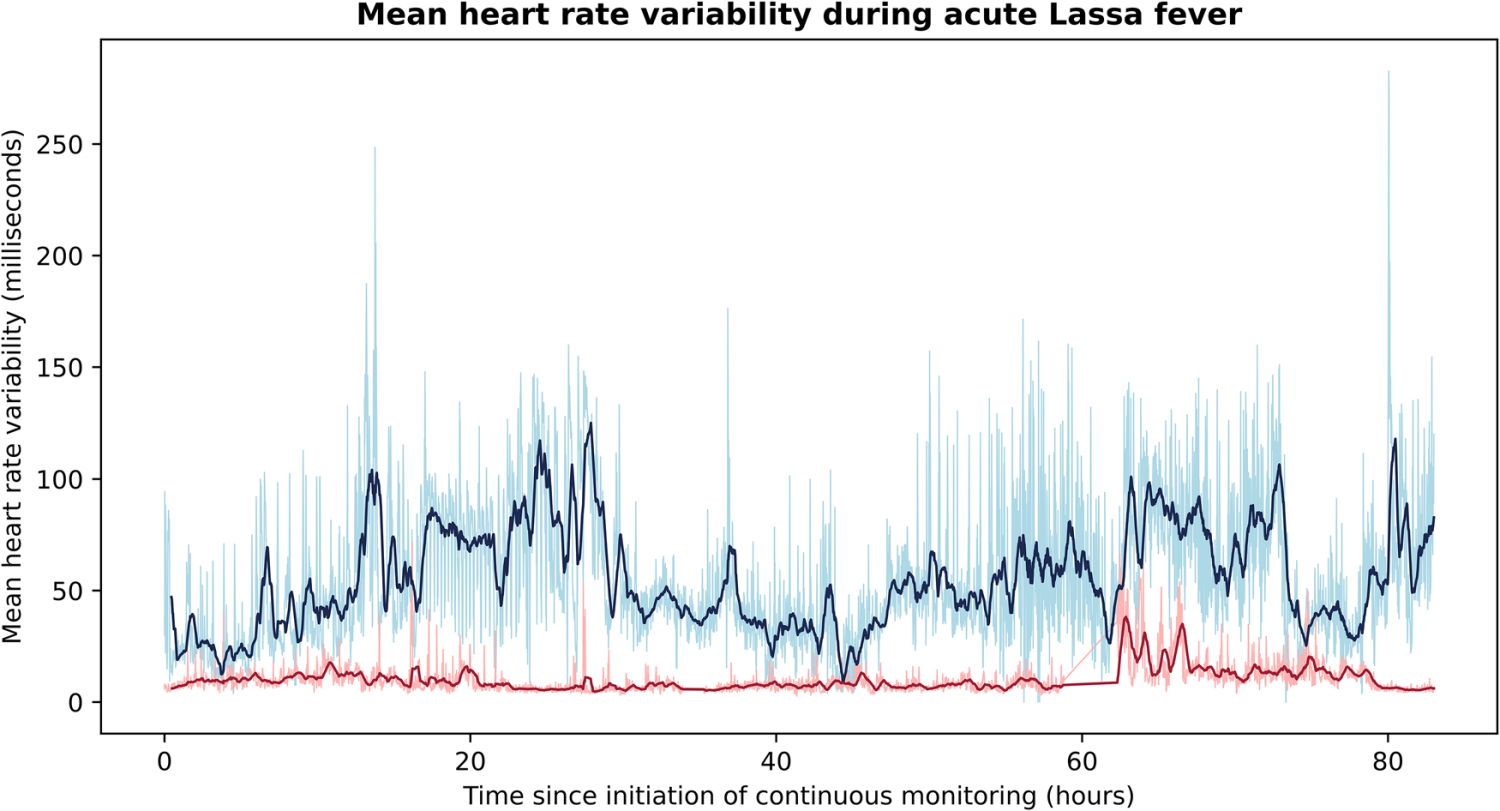
Mean heart rate variability during acute Lassa fever among survivors and the deceased. Time domain heart rate variability (HRV) is measured by the standard deviation from the mean time of RR intervals. The dark blue and dark red curves represent the 30-min rolling averages of vital signs for survivors (*n* = 3) and the deceased (*n* = 5), respectively, while the light blue and light red curves report vital signs at 1-min intervals. Among those that survived acute Lassa fever, mean HRV was higher than that of those who died at nearly all time points over the first 83 h of continuous monitoring. The mean HRV of survivors and the deceased was 59 and 10 ms, respectively.

Mean HRV among survivors was higher than that of those who died at nearly all time points during the continuous monitoring period (Fig. 6). The mean HRV of survivors and non-survivors over the entire course of their monitoring periods were 59 and 10 milliseconds (ms), respectively. The mean skin temperature for survivors was 35.9 °C, and for non-survivors was 36.5 °C.

**Fig. 7:**
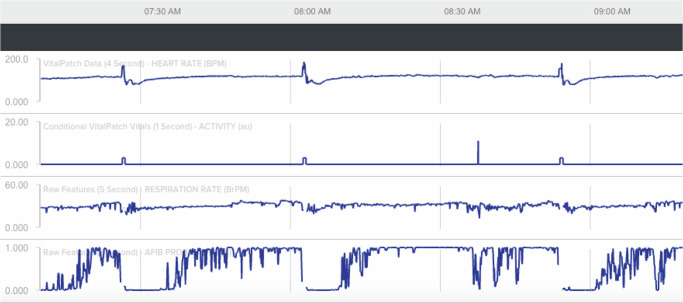
Analytic platform interpretation of waveforms produced from biosensor device data. An example of physiological parameters for Patient 4 changing in concert with one another. The patient has an elevated HR with a high probability for atrial fibrillation, as predicted by the analytics platform. The patient demonstrates several episodes of activity (positional changes from lying to sitting or standing), which initially increase the HR before lowering it and reducing the probability of atrial fibrillation to zero for periods of time. These interrelated findings may represent coughing fits with subsequent increases in vagal tone that suppressed atrial ectopy or ventricular responsiveness. Top waveform: heart rate (beats per minute). Second waveform: patient activity. Third waveform: respiratory rate (breaths per minute). Bottom waveform: probability of atrial fibrillation.

Some interesting physiological phenomena were captured by the biosensors, both in real-time on the Android monitoring devices and retrospectively on the analytics platform. For example, over the course of their monitoring, Patient 4 is generally tachycardic with several episodes of HR returning to normal range (Fig. 4). Visualization of the patient’s biosensor waveforms demonstrates the elevated HR with a high predicted probability of atrial fibrillation, which was due to frequent detection of ectopy (Fig. 7). The patient then experiences multiple episodes of activity, which initially increase the HR before lowering it and reducing the probability of atrial fibrillation to zero. This interplay of heart rate, arrhythmia, and patient activity suggests coughing fits with subsequent increases in vagal tone that temporarily suppress atrial ectopy or ventricular responsiveness, which was concomitantly observed by nursing on the Lassa ward^20^.

## Discussion

This is the first study evaluating the use of wearable biosensor devices for the continuous physiological monitoring of individuals with acute Lassa fever. Although this study was not powered to make conclusions about physiological parameters and their associations with clinical outcomes, we did observe that all but one patient who did not survive their illness had a mean HR in the age-specific tachycardic range, compared to none of the survivors. These findings are similar to what has been observed in similar settings for patients with bacterial sepsis^21^. Conversely, we found a less pronounced difference in mean RR between groups. It is worth noting that four of the five non-survivors were pediatric patients, while only one of the three survivors was a child, who are known to have higher resting HR and RR than adults^19^. We attempted to account for this by using established age-specific thresholds to define tachycardia and tachypnea. Elevations in respiratory rate during Lassa fever could be explained by several potential etiologies including encephalopathy, anxiety, hypoxemia, pleural effusion, acute anemia, lactic acidosis from impaired tissue perfusion, or acidosis from renal failure^22,23^.

There is robust clinical evidence that alterations in HRV correlate with severity of systemic infection and have prognostic value as a predictor of multiorgan dysfunction and death^11,24–26^. A normal HRV is between 19 and 75 ms. Though normal ranges for children have not been firmly established, it is believed that there is a linear relationship between HRV and age^27^. On average, individuals who survived acute Lassa fever demonstrated HRV within the normal range (mean HRV 59 ms), while those who died were characterized by HRV below the normal range (mean HRV 10 ms)^28^. Increases in HRV over the course of infection have also been shown to correlate with survival in ICU patients with septic shock^29^. In our study, non-survivors failed to mount any sustained increases in HRV. Pre-admission measurements for all patients were unavailable, which precluded the opportunity for comparison of HRV during acute illness with individuals’ healthy baselines. Still, results from our small sample suggest there may be a role for the assessment of HRV in Lassa fever prognostication that can be defined with further investigation.

The shaded gray areas in Fig. 4 represent periods of patient decompensation toward death that were captured by objective changes in vital signs, such as an acute increase in HR or RR. If used as real-time telemetry, the study’s monitoring system has the potential to identify these changes more rapidly than the current system of collecting patient vital signs every 30 min at the KGH Lassa ward, creating opportunities for earlier intervention and better outcomes^7^. This benefit may be even more pronounced in other regions of Lassa-endemic West Africa, where in-person patient monitoring by nursing is often more limited.

Further adoption of wearable biosensor devices to remotely monitor individuals with severe acute Lassa fever poses several challenges. First, we encountered recurrent lapses in patient data collection due to failure of the devices’ adhesive backing, which was indicated by elevations in impedance detected by the sensor. Temperatures during the dry season in Sierra Leone frequently surpass 38 °C with a high relative humidity^30^. These climatic conditions, along with poor ventilation in patient rooms in the Lassa ward, likely led to patient perspiration causing occasional adhesive failure on the devices.

Second, due to the floor plan of the Lassa ward at KGH, Android devices were occasionally out of range for the monitoring devices’ Bluetooth connectivity, leading to further lapses in patient data collection. This was addressed by situating the Android devices as close as possible to the patient area, though they could ultimately not be brought into the patient area due to the location of power outlets and concerns about infection control. However, even when both devices were within the Bluetooth radius of 10 meters, there were still occasional connectivity problems, an issue that was present in a previous study conducted in a Rwandan emergency department^7^.

Third, we observed several spurious vital sign readings that would be physiologically impossible, such as very extreme skin temperatures or respiratory rates of zero. These readings prompted us to discard patient data that was generated during the same 1-min window and raised concerns about rare but easily identifiable issues with device calibration. To explore whether profound hemodynamic shock may have affected skin temperature readings in our three adult participants, we reviewed manual vital signs at intervals when biosensor-reported skin temperatures were non-physiological. We found no temporal correlation between abnormal skin temperature reported by the biosensor and blood pressure as measured manually. We were unable to perform this same analysis for our pediatric participants, as there was no access to a pediatric sphygmomanometer at the KGH Lassa ward during the study period, and manual blood pressures were not collected for pediatric patients.

Fourth, at nearly US$170.00 each in 2024, the cost of wearable biosensor devices–not including the necessary Android devices to accompany them–may be prohibitive for many settings in which Lassa fever is endemic. Fifth, the VitalPatch device is intended for use on general care patients who are 18 years of age or older^31^. Although the device appears capable of collecting high-quality data from young children–as evidenced by the presence of 100% EKG signal quality from participants in our study as young as five months–we encountered issues with pediatric participants removing the device and disrupting the collection of data.

Lastly, Sierra Leone suffers from a profound shortage of clinicians, with only one physician per ten thousand population^32^. The country’s official Lassa ward does not have access to organ support equipment, such as mechanical ventilators or hemodialysis machines, and routine laboratory studies are performed infrequently. Even with the ability to identify patients at risk for death based on vital sign trends or acute decompensation, the burden on local physicians and the extreme scarcity of sufficient resources for meaningful intervention limits this technology from being implemented to its full potential.

Our study has several limitations. A more robust statistical analysis of our patients was limited by a small sample size, owing to the relative scarcity of diagnosed Lassa fever in Sierra Leone, which averaged just 8.4 cases annually from 2019 to 2023 (unpublished; from KGH records). Additionally, the majority (81.3%) of continuous physiological data we collected was ultimately discarded due to poor quality. There was also a bias toward younger individuals in our study, with the median age falling in the pediatric range. This is in accordance with existing epidemiologic data from Sierra Leone that suggest the highest incidence of antigenemic Lassa fever is observed in children and young adults^1^. The distribution of ages between survivors and non-survivors in our cohort limits the conclusions we can draw from our vital sign data, as younger patients on average have increased HR, increased RR, and decreased HRV compared to adults. Given our standard of 12 h of continuous high-quality data, participants who died in less than 12 h were not included in the analysis, which biased our results away from more imminently ill patients.

## Conclusions

Here we demonstrate that continuous vital signs monitoring of patients with Lassa fever in resource-limited settings can be challenging, with a substantial amount of data being of poor quality. Similar to what has been observed in bacterial sepsis, we found that HRV may be related to outcome in acute Lassa fever, though conclusions about HR and RR are confounded by age distributions within our cohort. Further research with a larger sample size is needed to determine whether trends of these physiologic parameters can be harnessed for Lassa fever prognostication. To improve the effectiveness of data collection using wearable devices in similar settings to Sierra Leone, efforts should be focused on the improvement of the adhesive in the context of patient perspiration and to ensure that the biosensor and Android devices are able to remain within Bluetooth range without breaches in infection control. If this can be achieved, then wearable biosensors may have a role in real-time remote patient monitoring of critically ill patients who pose an infectious risk in low-resource settings.

## Supplementary information

Supplementary information

Description of Additional Supplementary Files

Supplementary Data 1

Reporting Summary

## Data Availability

The Sierra Leone Ethics and Scientific Review Committee (SLESRC) under the Ministry of Health and Sanitation (MoHS) governs all human subject research conducted within the country and requires that raw data collected in Sierra Leone can be shared only with explicit written approval. Anonymized participant-level data and raw data generated during the current study will be made available upon reasonable request directed to the corresponding author. The source data underlying all figures can be found in Supplementary Data 1.
