# Supplementary material for: Continuous vital sign monitoring of acute Lassa fever using wearable biosensor devices in West Africa
Source: Commun Med (Lond). 2025 Jul 11;5:290. doi: 10.1038/s43856-025-01002-6 (PMC12254359; doi:10.1038/s43856-025-01002-6)
Supplement: Supplementary file 1 — Supplementary information [file 43856_2025_1002_MOESM1_ESM.pdf]

Group	Median age	Females	Mortality rate	Median time from presentation to death	Median time from enrollment to death	Total monitoring time
Included (n=8)	6 years	50%	62.5%	8 days	45 hours (17 – 271 hours)	788.5 hours
Excluded (n=9)	27 years	67%	66.7%	2 days	19 hours (8 – 69 hours)	483.7 hours
**Total** (n=17)	**21 years**	**59%**	**64.7%**	**2 days**	32 hours	1,272.2 hours

Supplementary Table 1. Demographic, clinical, and monitoring data for included and excluded individuals.
