# Supplementary material for: Continuous vital sign monitoring of acute Lassa fever using wearable biosensor devices in West Africa
Source: Commun Med (Lond). 2025 Jul 11;5:290. doi: 10.1038/s43856-025-01002-6 (PMC12254359; doi:10.1038/s43856-025-01002-6)
Supplement: Supplementary file 2 — Description of Additional Supplementary Files [file 43856_2025_1002_MOESM2_ESM.pdf]

Description of Additional Supplementary files

File name: Supplementary Data 1

Description: Supplementary data 1 contains the source data for all figures and tables.
