# Supplementary material for: Continuous vital sign monitoring of acute Lassa fever using wearable biosensor devices in West Africa
Source: Commun Med (Lond). 2025 Jul 11;5:290. doi: 10.1038/s43856-025-01002-6 (PMC12254359; doi:10.1038/s43856-025-01002-6)
Supplement: Supplementary file 4 — Reporting Summary [file 43856_2025_1002_MOESM4_ESM.pdf]

nature research

Corresponding author(s): Brady Page

Reporting Summary

Nature Research wishes to improve the reproducibility of the work that we publish. This form provides structure for consistency and transparency in reporting. For further information on Nature Research policies, see Authors & Referees and the Editorial Policy Checklist.

Statistical parameters

When statistical analyses are reported, confirm that the following items are present in the relevant location (e.g. figure legend, table legend, main text, or Methods section).

n/a	Confirmed	
☐	☒	The exact sample size (*n*) for each experimental group/condition, given as a discrete number and unit of measurement
☐	☒	An indication of whether measurements were taken from distinct samples or whether the same sample was measured repeatedly
☒	☐	The statistical test(s) used AND whether they are one- or two-sided<br>*Only common tests should be described solely by name; describe more complex techniques in the Methods section.*
☐	☒	A description of all covariates tested
☒	☐	A description of any assumptions or corrections, such as tests of normality and adjustment for multiple comparisons
☐	☒	A full description of the statistics including central tendency (e.g. means) or other basic estimates (e.g. regression coefficient) AND variation (e.g. standard deviation) or associated estimates of uncertainty (e.g. confidence intervals)
☒	☐	For null hypothesis testing, the test statistic (e.g. *F*, *t*, *r*) with confidence intervals, effect sizes, degrees of freedom and *P* value noted<br>*Give P values as exact values whenever suitable.*
☒	☐	For Bayesian analysis, information on the choice of priors and Markov chain Monte Carlo settings
☒	☐	For hierarchical and complex designs, identification of the appropriate level for tests and full reporting of outcomes
☒	☐	Estimates of effect sizes (e.g. Cohen's *d*, Pearson's *r*), indicating how they were calculated
☒	☐	Clearly defined error bars<br>*State explicitly what error bars represent (e.g. SD, SE, CI)*

Our web collection on statistics for biologists may be useful.

Software and code

Policy information about availability of computer code

Data collection	No software was used.
Data analysis	Python language was used to create custom script for performing descriptive statistics in this analysis.

For manuscripts utilizing custom algorithms or software that are central to the research but not yet described in published literature, software must be made available to editors/reviewers upon request. We strongly encourage code deposition in a community repository (e.g. GitHub). See the Nature Research guidelines for submitting code & software for further information.

Data

Policy information about availability of data

All manuscripts must include a data availability statement. This statement should provide the following information, where applicable:

- Accession codes, unique identifiers, or web links for publicly available datasets
- A list of figures that have associated raw data
- A description of any restrictions on data availability

The data generated and analyzed in this study are available upon reasonable request from the corresponding author.
Due to ethical and privacy concerns, some data cannot be publicly shared. Researchers seeking access to restricted data must submit a formal request to the Scripps

Research institutional review board (IRB) and comply with applicable data use agreements.
Custom scripts and code used for data analysis are available upon request.

Field-specific reporting

Please select the best fit for your research. If you are not sure, read the appropriate sections before making your selection.

☒ Life sciences ☐ Behavioural & social sciences

For a reference copy of the document with all sections, see nature.com/authors/policies/ReportingSummary-flat.pdf

Life sciences

Study design

All studies must disclose on these points even when the disclosure is negative.

Sample size	Sample size calculations were not performed. Instead, all eligible and consenting individuals present during the enrollment period were eligible for inclusion.
Data exclusions	Waveform data from the EKG leads, thermistor, and accelerometer present on biosensor devices were analyzed by the machine learning algorithms of the physIQ platform to generate the following parameters at one-minute intervals: skin temperature, heart rate (HR), respiratory rate (RR), time domain heart rate variability (HRV), and EKG signal quality. The physIQ algorithm makes calls to the biosensor at one-minute intervals via a Python middleware script that allows for the collection of biosensor data, as previously described12,17. The algorithm is applied to the EKG signal to assess the signal's strength, motion artifact content, and noise levels over the one-minute intervals. This results in a quality metric that ranges from 0 to 100% for each one-minute data window. If the metric falls below 75%, the corresponding one-minute data window is automatically rejected for use in calculating vital signs from the raw biosignal data. As a quality control measure, and since the focus of the study was on physiological trends throughout the course of Lassa fever hospitalization, we established standards for the EKG signal quality metric and vital sign values. In addition to the automatic filtering of patient physiological data to remove one-minute data windows where EKG quality was deemed by the machine learning algorithms to be less than 75%, we also filtered one-minute data windows during which vital sign readings were physiologically unlikely–namely, skin temperature < 33°C or > 42°C, or respiratory rate of 0. Patients who did not produce at least twelve hours of continuous high-quality physiological monitoring data were not included in the analysis.
Replication	As each patient would present unique vital sign data, the question of reproducibility would not specifically apply to this investigation.
Randomization	There was no randomization in our study, as there was no intervention.
Blinding	There was no blinding in our study, as there was no intervention.

Materials & experimental systems

Policy information about availability of materials

n/a	Involved in the study
☒	☐ Unique materials
☒	☐ Antibodies
☒	☐ Eukaryotic cell lines
☒	☐ Research animals
☐	☒ Human research participants

Human research participants

Policy information about studies involving human research participants

Population characteristics	Individuals of all ages that presented to the study site with confirmed Lassa fever were eligible for enrollment.

Method-specific reporting

n/a	Involved in the study
☒	☐ ChIP-seq
☒	☐ Flow cytometry
☒	☐ Magnetic resonance imaging
